# Changes and inequalities in early birth registration and childhood care and education in Vietnam: findings from the Multiple Indicator Cluster Surveys, 2006 and 2011

**DOI:** 10.3402/gha.v9.29470

**Published:** 2016-02-29

**Authors:** Kim Bao Giang, Juhwan Oh, Vu Duy Kien, Luu Ngoc Hoat, Sugy Choi, Chul Ou Lee, Hoang Van Minh

**Affiliations:** 1 Department of Health Education and Center for Health System Research, Hanoi Medical University, Hanoi, Vietnam; 2 JW LEE Center for Global Medicine, Seoul National University College of Medicine, Seoul, Korea; 3 Center for Population Health Sciences, Hanoi School of Public Health, Hanoi, Vietnam; 4 Department of Biostatistics and Health Informatics, Hanoi Medical University, Hanoi, Vietnam

**Keywords:** inequality, birth record, school admission, childhood care, childhood education

## Abstract

**Introduction:**

Early birth registration, childhood care, and education are essential rights for children and are important for their development and education. This study investigates changes and socioeconomic inequalities in early birth registration and indicators of care and education in children aged under 5 years in Vietnam.

**Design:**

The analyses reported here used data from the Vietnam Multiple Indicator Cluster Surveys (MICS) in 2006 and 2011. The sample sizes in 2006 and 2011 were 2,680 and 3,678 for children under 5 years of age. Four indicators of childcare and preschool education were measured: birth registration, possession of books, preschool education attendance, and parental support for early childhood education. The concentration index (CI) was used to measure inequalities in gender, maternal education, geographical area, place of residence, ethnicity, and household wealth.

**Results:**

There were some improvements in birth registration (86.4% in 2006; 93.8% in 2011), preschool education attendance (57.1% in 2006; 71.9% in 2011), and parental support for early childhood education (68.9 and 76.8%, respectively). However, the possession of books was lower (24.7% in 2006; 19.6% in 2011) and became more unequal over time (i.e. CI=0.370 in 2006; CI=0.443 in 2011 in wealth inequality). Inequalities in the care and education of children were still persistent. The largest inequalities were for household wealth and rural versus urban areas.

**Conclusion:**

Although there have been some improvements in this area, inequalities still exist. Policy efforts in Vietnam should be directed towards closing the gap between different socioeconomic groups for the care and education of children under 5 years old.

## Introduction

According to the United Nations (UN) Convention on the Rights of the Child, a child should have the right to be registered at birth, be cared for by his or her parents, and have access to services such as education and health care (1). Birth registration records have been acknowledged as ‘passports to protection’ (2). It is well documented that the first few years of a child's life are vital for their health and social development and are also important in terms of contributing to the establishment of a country's human capital (3, 4). However, according to a 2013 report by the UN Children's Fund (UNICEF), there were 230 million unrecorded child births worldwide, and the global birth registration rate was 65%. In South Asia, the birth registration rate was only 39% (5).

Apart from giving children a name and getting a birth certificate, parents and guardians are expected to support their children's development by providing them with appropriate care and education (6). However, an earlier report by UNICEF in 2012 indicated that more than half of the children from two-thirds of the 30 countries that conducted the Multiple Indicator Cluster Surveys (MICS) had fewer than three books in their homes. In one-third of these countries, less than 10% of children above 3 years of age did not receive early childhood care and education (7). The two UNICEF reports highlight the need for countries to increase their investments in early childhood development and address inequalities in childcare and protection. Children from poor households, those living in ‘informal settlements’ or slums, and children whose parents have low education are less likely to receive the care and education needed for their development (7, 8).

Vietnam was the first country in Asia, and the second country worldwide, to ratify the 1990 UN Convention on the Rights of the Child (9). As a result of efforts rendered by the Vietnamese government during the past two decades, life has improved for 26 million Vietnamese children. Most of the children in Vietnam now have access to adequate health care and can expect to live longer than their parents. In addition, most children in Vietnam can attend primary and secondary school. According to UNICEF, the out-of-school rate among primary school–aged children in Vietnam declined from 9.7% in 2005 to 1.9% in 2013 (10).

Yet, as in many other countries, inequalities are persistent in Vietnam (11). In 2006, 40% of children living in rural areas were poor, compared with about 10% of children living in urban areas. Geographical disparities persist in education; about 75% of urban children attend preschools, compared with only 51% of children in rural areas (12). The proportion of ethnic minority children who completed the first 5 years of primary education was just over 60%, compared with 86% of ethnic majority children (13). In addition, the school dropout rate was higher among ethnic minority households, compared with households from ethnic majority groups (50% vs. 16%) due to school fees, language barriers, and travel constraints (14). Yet, to our knowledge, no studies to date have investigated trends in childcare and education inequalities and inequities among children under 5 years in Vietnam. This paper investigates changes and socioeconomic inequalities in birth registration and early childhood care and education in children under 5 years of age in Vietnam between 2006 and 2011.

## Methods

### Data source

This study used data from the Vietnam MICS in 2006 and 2011. The MICS are household surveys supported by UNICEF. They began in 1995 (15). The objectives of the MICS were to ‘provide up-to-date information for assessing the situation of children and women and generate data for the identification of vulnerable groups, inequities and disparities, as a basis for informing policies and interventions’. The MICS were designed to provide health and other indicators on children and women at the national level, in both urban and rural areas in six geographical regions of Vietnam. Two-stage sampling was used to select households for participation in the surveys. The main sampling strata in each region were urban and rural areas. Within each stratum, a specific number of enumeration areas were identified using the probability proportional to size method. In each enumeration, 20 households were systematically selected using an updated list of households. The MICS administered three questionnaires to collect information on households (number of members, dwelling characteristics, etc.), women aged 15–49 years in the selected households, and children under 5 years in the same households, by interviewing mothers or caregivers.

More than 100 countries have participated in the MICS. The children's questionnaire collected information about different aspects of childhood development, including health, nutrition, education, child protection, and HIV/AIDS. Early childhood development indicators, designed to measure access to early childhood care and education outside the home, were introduced to the MICS in the third round in 2006.

In Vietnam, the MICS were conducted by the General Statistics Office in collaboration with the Ministry of Health and the Ministry of Labor, Invalids and Social Affairs, with financial and technical supports from UNICEF and the UN Population Fund. The sample sizes for children under 5 years of age in the 2006 and 2011 MICS were 2,680 and 3,678, respectively.

### Dependent variables

The following indicators of birth registration and early childhood care and education for children aged 5 years and younger were used to derive the four binary dependent variables described below.

- *Birth registration*: Included all children under 5 years old who either possessed a birth certificate or were registered with the relevant civil authority.
- *Possession of books*: Having at least three books, including picture books, designated for children under 5 years old in the home.
- *Preschool education attendance*: Attendance at organized learning or early childhood education programs run by a private or government facility, including kindergarten or community childcare services. This information was collected only among children 3–5 years of age.
- *
Support of parents*: Engagement of parents in promoting learning and preparing children for schools (3–5 years of age). The parents were considered to be engaged if during the 3 days prior to the interview, any family member over 15 years of age had been engaged in four or more of the following activities with the 3–5-year-old child in the family: 1) reading book(s) or looking at the picture book(s) with the child; 2) telling a story to the child; 3) singing song(s), including lullabies, to or with their child; 4) taking the child outside the home, compound, yard, or enclosure; 5) playing with the child; 6) naming, counting, or drawing things with the child.

### Independent variables

The explanatory variables are as follows:

1. Sex of the child.
2. Ethnicity of the head of household. This is an important factor because the head of household often has decisive role in the health and education of the household members, and attitudes in this regard vary with ethnic groups and cultures (16). The Kinh group is the largest ethnic group in Vietnam.
3. Mother's education was classified using the current classification for the Vietnamese education system.
4. Household wealth status was measured by asset-based quintiles.
5. Living region of the household was one of six geographical regions within Vietnam (Red River Delta, Northern Midlands and Mountain Areas, Northern Central and Coastal Areas, Central Highlands, South East, and Mekong River Delta). Each region has several provinces and covers both rural and urban areas.
6. Place of residence was defined as *rural* or *urban*.
7. The year of the survey was 2006 or 2011.

### Statistical analyses

Both descriptive and analytical statistics were used. Individual and household characteristics of the study samples (2006 and 2011) are described. The proportion of children who met each of the four childhood indicators (birth registration, books, preschool education, and parental support) was estimated for 2006 and 2011. The two survey years were compared using the Wald test. Crude odds ratios for each of the indicators were estimated by socioeconomic subgroups. Socioeconomic inequalities are described using the following: 1) concentration indices (CIs) and 2) adjusted odds ratios from the logistic regression models for each of the four childhood indicators. The CI is an indicator used to measure the magnitude of socioeconomic inequality with regard to a variable of interest. In this study, the CI indicates the extent to which a childcare indicator is concentrated among the disadvantaged or the advantaged. A higher absolute value of CI demonstrates a greater level of inequality (17–19). Four socioeconomic inequality measures based on the method by Wagstaff et al. (18) were used. These measures are gender, educational attainment, ethnicity, urbanity, and wealth (17).

Survey sampling population weights were used in all analyses. A significance level of *p*<0.05 was applied. STATA version 12 was used for the data analyses.

### Results

Household and individual characteristics are described for the 2006 and 2011 study samples and for the combined samples (overall). Table 1 shows that overall among children under 5 years of age there were more male (51.3%) than female children (48.7%) and most children belonged to Kinh households (84.1%). The proportion of children whose mothers had primary education or less was 36.0% in 2006 and 41.6% in 2011. The proportion of mothers with upper high school education was lower in 2011 compared with 2006 (18.2% vs. 51.1%) (Table 1).

**Table 1:** Individual and household characteristics of children under 5 years, Vietnam, MICS, 2006 and 2011

	2006		2011		Overall	
			
	*n*	%	*n*	%	*n*	%
Age group of the child (months)						
<12	483	18.0	668	18.2	1,151	18.1
12–35	1,115	41.6	1,551	42.2	2,666	41.9
36–48	615	23.0	833	22.6	1,448	22.8
49–60	467	17.4	626	17.0	1,093	17.2
Sex of the child						
Male	1,394	52.0	1,869	50.8	3,263	51.3
Female	1,286	48.0	1,809	49.2	3,095	48.7
Ethnicity of household head						
Kinh	2,205	82.3	3,143	85.5	5,348	84.1
Other	475	17.7	535	14.5	1,010	15.9
Mother's education						
Less than primary level	965	36.0	1,529	41.6	2,494	39.2
Lower secondary level	344	12.9	1,479	40.2	1,823	28.7
Upper secondary level	1,371	51.1	670	18.2	2,041	32.1
Wealth quintile						
Poorest	542	20.2	831	22.6	1,372	21.6
Second poorest	466	17.4	673	18.3	1,139	17.9
Middle	549	20.5	700	19.0	1,250	19.7
Second richest	555	20.7	749	20.4	1,304	20.5
Richest	568	21.2	725	19.7	1,293	20.3
Region						
Red River Delta	515	19.2	798	21.7	1,312	20.6
Northern Midlands and Mountain Areas	454	16.9	707	19.2	1,161	18.3
Northern Central and Coastal Areas	574	21.4	719	19.5	1,293	20.3
Central Highlands	139	5.2	233	6.3	373	5.9
Southeast	440	16.4	572	15.6	1,011	15.9
Mekong River Delta	559	20.8	650	17.7	1,208	19.0
Area						
Urban	633	23.6	1,013	27.5	1,646	25.9
Rural	2,047	76.4	2,665	72.5	4,712	74.1

MICS, Multiple Indicator Cluster Surveys.

Early childcare and education indicators generally improved between 2006 and 2011, except for the indicator *having at least three books* (Table 2). In 2011, the proportion of children under 5 years with birth registration records was 93.8%. This indicator improved between the surveys, and the difference between 2011 and 2006 was 7.4% (95% confidence interval [CI]: 5.7–9.1%). The proportion of children under 5 years of age having at least three children's books declined from 24.7% in 2006 to 19.6% in 2011. Preschool education attendance rates and parental support in childcare and educational activities among children from 3 to 5 years old increased between 2006 and 2011. Preschool education attendance increased from 57.1% in 2006 to 71.9% in 2011, and the difference was 14.8%. The proportion of children with parental support for their childcare and educational support rose from 68.9% in 2006 to 76.8% in 2011 (Table 2).

**Table 2:** Comparison of early childhood indicators, Vietnam, MICS, 2006 and 2011

	2006		2011		Difference between 2011 and 2006	
			
Variables	%	95% CI	%	95% CI	Difference (%)	95% CI
Children 0–4 years old						
Birth registration	86.4	85.0–87.9	93.8	93.0–94.7	7.4	5.7–9.1
Having at least three books	24.7	22.8–26.5	19.6	18.1–21.1	–5.1	(–7.4)–(–2.7)
Children 3 to ≤5 years old						
Preschool education attendance	57.1	53.8–60.4	71.9	69.3–74.6	14.8	10.7–22.4
Parental support for learning	68.9	65.8–72	76.8	74.2–79.4	7.9	3.8–11.9

CI, concentration index; MICS, Multiple Indicator Cluster Surveys.

The results from logistic regression models (Table 3) show that, compared with 2006, in 2011 children were more likely to have birth registration, preschool education attendance, and parental support. However, in 2011 children were less likely to have at least three books at home than they were in 2006 (odds ratio [OR]=0.8).

**Table 3:** Logistic regression of early childhood indicators, Vietnam, MICS, 2006 and 2011

	Birth registration		Having at least three books		Preschool education attendance		Parental support	
				
	OR	95% CI	OR	95% CI	OR	95% CI	OR	95% CI
MICS (2006)								
2011	2.7	2.3–3.2	0.7	0.7–0.8	1.9	1.6–2.3	1.5	1.3–1.8
Age group (<12 months)								
12–35 months	3.3	2.6–4.1	2.9	2.2–3.8	NA		NA	
36–47 months	3.8	2.9–5.1	4.9	3.7–6.5	1		1	
48–59 months	5.8	4.1–8.3	6.5	4.9–8.7	2.9	2.4–3.5	0.9	0.8–1.2
Sex (male)								
Female	1.1	0.9–1.2	0.9	0.8–1.0	1.2	1.01–1.4	1.1	0.9–1.3
Mother's education (primary or less)								
Lower secondary	2.1	1.6–2.7	0.5	0.4–0.6	1.1	0.9–1.4	1.0	0.8–1.2
Upper secondary	1.6	1.3–2.0	1.3	1.1–1.5	1.2	1–1.5	1.6	1.3–2.1
Ethnicity (other)								
Kinh	6.6	5.5–7.8	4.7	3.8–5.8	2.1	1.7–2.5	2.8	2.3–3.5
Area (rural)								
Urban	3.9	3.1–4.9	3.9	3.4–4.5	2.4	1.9–2.9	2.8	2.2–3.4
Region (Red River Delta)								
Northern Midlands and Mountain Areas	0.2	0.1–0.3	0.3	0.2–0.4	0.4	0.3–0.6	0.4	0.3–0.6
North Central and Coastal Areas	0.2	0.1–0.4	0.5	0.4–0.6	0.3	0.2–0.4	0.3	0.2–0.4
Central Highlands	0.1	0.1–0.2	0.4	0.3–0.4	0.2	0.1–0.3	0.2	0.1–0.3
South East	0.5	0.3–0.9	1.1	0.9–1.3	0.3	0.2–0.5	0.3	0.2–0.5
Mekong River Delta	0.1	0.1–0.2	0.3	0.3–0.4	0.1	0.1–0.2	0.1	0.1–0.2
Wealth quintile (poorest)								
2nd quintile	2.4	1.9–3.1	2.0	1.4–2.9	1.4	1–1.8	1.3	1–1.7
3rd quintile	4.3	3.2–5.9	4.3	3.1–6.1	2.0	1.5–2.6	1.8	1.3–2.4
4th quintile	5.0	3.6–6.9	7.0	5.1–9.6	2.5	1.9–3.3	2.1	1.5–2.8
5th quintile	10.1	6.5–15.5	23.8	17.5–32.5	6.2	4.4–8.7	6.6	4.5–9.8

Note: Reference categories are in parentheses; NA, not applicable; MICS, Multiple Indicator Cluster Surveys.

**Table 4:** Concentration indices for early childhood indicators, Vietnam, MICS, 2006 and 2011

	Birth registration		Having at least three books		Preschool education attendance		Parental support	
				
Survey year	CI	Lower and upper bounds	CI	Lower and upper bounds	CI	Lower and upper bounds	CI	Lower and upper bounds
Sex (male)								
2006	−0.009	−0.019; 0.000	−0.089	−0.134; −0.044	−0.011	−0.046; 0.023	−0.009	−0.035; 0.017
2011	−0.002	−0.007; 0.003	−0.012	−0.056; 0.032	−0.049	−0.071; −0.028	0.019	0.000; 0.039
Mother's education (primary or less)								
2006	0.009	−0.001; 0.019	0.023	−0.021; 0.066	−0.010	−0.045; 0.024	0.022	−0.005; 0.049
2011	0.012	0.007; 0.018	−0.022	−0.071; 0.027	−0.005	−0.026; 0.015	0.043	0.025; 0.061
Ethnicity (minority)								
2006	0.017	0.006; 0.029	0.011	−0.03; 0.053	−0.027	−0.064; 0.009	0.012	−0.016; 0.041
2011	0.008	0.001; 0.014	0.061	0.02; 0.103	−0.081	−0.102; −0.059	0.020	0.000; 0.039
Area (rural)								
2006	0.005	−0.004; 0.014	**0.201**	0.152; 0.250	0.008	−0.027; 0.043	**0.057**	**0.030; 0.083**
2011	0.001	−0.004; 0.006	**0.210**	0.164; 0.256	−0.062	−0.081; −0.044	0.032	0.013; 0.051
Wealth (poorest)								
2006	**0.054**	0.044; 0.064	**0.370**	0.334; 0.405	**0.133**	0.101; 0.165	**0.082**	0.057; 0.107
2011	0.025	0.019; 0.031	**0.443**	0.407; 0.479	**0.060**	0.04; 0.08	**0.074**	0.055; 0.093

Note: Reference categories are in parentheses; MICS, Multiple Indicator Cluster Surveys. Bold values are for CIs of greater than 0.05 and statistically significant.

The older children had a higher likelihood of being registered, having at least three children's books, and attending preschool. Compared with children less than 12 months, children 49–59 months were more likely to be registered (OR=8.4) and to have at least three books at home (OR=11.6), respectively. Children of 49–59 months were more likely to attend a preschool education program compared with those aged 36–48 months (OR=2.9).

Gender was not a significant predictor on any of the four indicators. Compared with children from minority households, children from Kinh households were more likely to be registered, have at least three books, attend a preschool education program, and have parental support. Compared with children from households in rural areas, children from households in urban areas were more likely to own at least three books (OR= 3.9: 95% CI 3.4–4.5) and to have parental support for their learning (OR= 2.4: 95% CI 2.2–3.4).

The geographical distribution of the four indicators varied. Compared to children living in the Red River Delta, the children in the other five regions were less likely to be registered, own at least three books, attend preschool education, and receive parental support.

Household wealth was strongly associated with all four indicators. As household wealth increased, so did the likelihood of having birth registration, at least three books, preschool education attendance, and parental support. Compared with the children in the poorest wealth quintile, the odds of being registered, having at least three books, attending preschool education, and having parental support were higher for children in the wealthiest quintile.

Inequalities in childcare and education measured by CI generally improved over time. In 2006, inequality in birth registration was highest by household wealth (CI=0.054), followed by ethnicity (CI=0.017). The CIs for having at least three books show that inequality was highest for wealth (CI=0.370), followed by area (CI=0.201). Inequality in preschool attendance rates was largest for wealth quintiles (CI=0.133). Inequality in parental support was also found to be largest for wealth (CI=0.082), followed by area (CI=0.057).

The comparisons between CIs in 2006 and 2011 indicate that CIs for birth registration between socioeconomic groups were declined but the significant reduction in inequality was only found for wealth. For possession of books, the inequality increased for ethnicity, sex, area, and household wealth. However, the increased inequality was only significant for wealth (CI=0.443 in 2011 vs. CI =0.370 in 2006). For inequality in preschool attendance, there were significant reductions for ethnicity, area, and wealth. The inequality in parental support decreased in 2011 but was not statistically significant (Table 4).

## Discussion

Our study investigated patterns in childhood indicators for care and education in Vietnam using data collected in national surveys conducted in Vietnam in 2006 and 2011. The results showed that Vietnam has achieved improvements in three early childhood indicators – birth registration, preschool registration, and parental support for learning – but not for the possession of books in the home. Similarly, inequalities in birth registration, preschool registration, and parental support for learning decreased, but inequalities increased for the availability of children's books. The largest inequalities were for household wealth, area (urban or rural), and ethnic group (Kinh or non-Kinh).

The 2011 MICS provides the most recent data on under 5 years old early childhood indicators in Vietnam. The results of this study showed that in 2011 the birth registration rate was 94%, preschool education attendance among children aged 3–5 years was 72%, parental support was 77%, and the proportion of children having at least three children's books in the home was 20%. The birth registration rate is comparable with other countries. For example, according to UNICEF report in 2012 (2), birth registration rates ranged from 39% in South Asia to 98% in Central and Eastern Europe. The Vietnamese birth registration rate was much higher than that reported in Laos (5% in 2011) (20), but much lower than in Thailand (42.7% in 2012) (21) and North Korea (79.1% in 2011) (22).

The preschool attendance rate among children aged between 3 and 5 years in Vietnam (71.9%) was higher than that in Laos (23%) (20) and Indonesia (38.9%) (23), whereas it was lower than in Thailand (84.4%) (21) and North Korea (97.8%) (22). The proportion of children who had parental support for their learning in Vietnam (76.8% in 2011) was lower than reported in North Korea in 2009 (90.8%) (22) and in Thailand in 2012 (92.7%) (21), although higher than in Laos in 2011 (57.4%) (20). The proportion of children having more than three children's books (19.6%) was much higher than in Laos (5% in 2011), but lower than in Thailand (42.7% in 2011) and North Korea (79.1% in 2011).

The improvement in the childhood indicators can be partly explained by a number of child development policies and programs implemented in Vietnam during recent decades. For example, the 2004 Vietnamese Law on Child Protection, Care, and Education and Decree 71/2011 provide the legal framework and guidance and responsibilities for different agencies and institutions in relation to child protection and care in Vietnam (24, 25). Since the introduction of this law in 2004, Vietnam has instituted many other supportive policies and programs (26). However, the proportion of children under 5 years having at least three children's books at home declined by 5%, while inequality across household wealth quintiles persisted over time. These problems can be explained by the lack of attention given to the provision of books or by the absence of measures to improve the affordability of children's books. Alternatively, the development of modern communication such as smartphones, TV programs, and other technological mediums may, in part, explain the reduction in use of printed books in recent years.

Although there were some improvements in terms of inequalities and changes in average levels, disparities between socioeconomic groups for these four childhood indicators were evident. Consistently, household wealth status and ethnicity were the most significant predictors of childcare and education in Vietnam. The most disadvantaged groups were from ethnic minorities or poorer households, mothers with lower educational levels, or from the Mekong River Delta and Southeast Regions. These trends are consistent with the results of studies conducted in previous years (7, 8, 27). However it should be noted that the gender difference in childcare and education was not significant. This can, in part, be attributed to the successful implementation of laws and policies related to child and gender equity in Vietnam (24, 28). The CI in 2011 indicates that the largest inequality was found in the availability of children's books at home within household wealth quintiles (0.443), followed by area of residence (0.21). These two CIs suggest high inequalities (17), with a skewed availability of children's books, mostly concentrated among rich households in urban areas. Furthermore, although inequalities in birth registration and preschool education attendance tended to decline among almost all socioeconomic subgroups from 2006 to 2011, the inequality in the availability of children's books in homes significantly increased by household wealth quintiles (from 0.37 to 0.443). This implies that the gap between the rich and poor became substantial. This finding is considered critical because several policies have been directed towards supporting childcare and education among the poor in Vietnam during the last decade. However, the need for increasing the availability of children's books has not attracted the attention of policy makers to the same extent as other childhood indicators.

### Strengths and limitations

This is an important study and the first of its kind in Vietnam. The findings are important for policy making in relation to early childhood education and care. However, they should be interpreted with caution, as there are some limitations. First, the analyses do not demonstrate a causal relationship because the study was conducted based on data from a repeated cross-sectional survey instead of data from a linked follow-up longitudinal survey. Second, due to limited information such as the lack of area-level database in the MICS, we were unable to integrate other significant variables into this analysis that might have influenced the results; for instance, the availability of preschool education in the subjects’ area (i.e. bookstore, preschool institution, etc.) was not included. Lastly, even though we investigated four important outcomes, they were not sufficient to measure all aspects of education and childcare during the preschool period. However, taking separate phases into account in terms of childhood development needs was beyond the scope of this study. There are many possibilities for future study designs to inform policy in this area. Longitudinal cohort studies, for example, can provide insights into associations that are not possible with cross-sectional designs.

## Conclusions

There have been some improvements in childcare and preschool education indicators in Vietnam for birth registration, preschool attendance, and parental support for learning. Socioeconomic inequalities have also decreased for these indicators. However, it is of concern that the availability of books has decreased and socioeconomic inequalities have widened. These findings draw attention to the need for policies to increase the availability of books for young children and to provide all Vietnamese children with equal educational opportunities, regardless of their socioeconomic status.
